# Impact of patient education on chronic heart failure in primary care (ETIC): a cluster randomised trial

**DOI:** 10.1186/s12875-016-0473-4

**Published:** 2016-07-19

**Authors:** Hélène Vaillant-Roussel, Catherine Laporte, Bruno Pereira, Marion De Rosa, Bénédicte Eschalier, Charles Vorilhon, Romain Eschalier, Gilles Clément, Denis Pouchain, Jean-François Chenot, Claude Dubray, Philippe Vorilhon

**Affiliations:** General Practice Department, Faculty of Medicine of Clermont-Ferrand University, 28 Place Henri Dunant, 63000 Clermont-Ferrand, France; Clinical Investigation Center, INSERM CIC 1401, Clermont-Ferrand University Hospital, 58 Rue Montalembert, 63000 Clermont-Ferrand, France; EA 7280 NPsy-Sydo, Faculty of Medicine of Clermont-Ferrand, University of Auvergne, 28 Place Henri Dunant, 63000 Clermont-Ferrand, France; Biostatistics unit, Clinical Research and Innovation Department, Clermont-Ferrand University Hospital, 58 Rue Montalembert, 63000 Clermont-Ferrand, France; Cardiology Department, Clermont-Ferrand University Hospital, 58 Rue Montalembert, 63000 Clermont-Ferrand, France; General Practice Department, Faculty of Medicine of Tours University, 10 boulevard Tonnellé, 37032 Tours, France; Department of General Practice, Institute of Community Medicine, University of Greifswald, Fleischmannstr. 42-44, 17475 Greifswald, Germany; Clermont University, University of Auvergne, EA 4681, PEPRADE (Périnatalité, grossesse, Environnement, PRAtiques médicales et DEveloppement), Clermont-Ferrand, France

**Keywords:** Heart failure, Primary care, Quality of life, Patient education, Cluster randomised controlled trial

## Abstract

**Background:**

The *Education Thérapeutique des patients Insuffisants Cardiaques* (ETIC; Therapeutic Education for Patients with Cardiac Failure) trial aimed to determine whether a pragmatic education intervention in general practice could improve the quality of life of patients with chronic heart failure (CHF) compared with routine care.

**Results:**

This cluster randomised controlled clinical trial included 241 patients with CHF attending 54 general practitioners (GPs) in France and involved 19 months of follow-up. The GPs in the Intervention Group were trained during a 2-day interactive workshop to provide a patient education programme. The mean age of the patients was 74 years (±10.5), 62 % were men and their mean left-ventricular ejection fraction was 49.3 % (± 14.3). At the end of the follow-up period, the mean Minnesota Living with Heart Failure Questionnaire scores in the Intervention and Control Groups were 33.4 (± 22.1) versus 27.2 (± 23.3; *P* = 0.74, intra-cluster coefficient [ICC] = 0.11). At the end of the follow-up period, the 36-Item Short Form Health Survey (mental health and physical health) scores in the Intervention and Control Groups were 58 (± 22.1) versus 58.7 (± 23.9; *P* = 0.58, ICC = 0.01) and 52.8 (± 23.8) versus 51.6 (± 25.5; *P* = 0.57, ICC = 0.01), respectively.

**Conclusions:**

Patient education delivered by GPs to elderly patients with stable heart failure in the ETIC programme did not achieve an improvement in their quality of life compared with routine care. Further research on improving the quality of life and clinical outcomes of elderly patients with CHF in primary care is necessary.

**Trial registration:**

The *Education Thérapeutique des patients Insuffisants Cardiaques* (*ETIC;* Therapeutic Education for Patients with Cardiac Failure) trial is a cluster randomised controlled trial registered with ClinicalTrials.gov (Registration Number: NCT01065142) and the French Drug Agency (*Agence Nationale de Sécurité du Médicament et des Produits de Santé*; Registration Number: 2009-A01142-55).

**Electronic supplementary material:**

The online version of this article (doi:10.1186/s12875-016-0473-4) contains supplementary material, which is available to authorized users.

## Background

Chronic heart failure (CHF) is a common condition that is increasing in prevalence with the ageing of the population and with improvements in the management of acute and chronic heart disease, especially ischaemic cardiomyopathies [1]. The prevalence of CHF in French general practice is estimated to be about 10 % for patients aged over 59 years [2]. The European Society of Cardiology guidelines recommend medical and electrical management to reduce morbidity and mortality and improve quality of life. They also recommend non-pharmacological management including self-care management, patient education, and self-care behaviour to improve patients’ adherence to treatment and quality of life [3].

In France, patient education programmes delivered by multidisciplinary teams in outpatient clinics attached to hospitals have been assessed for their impact on rehospitalisation, mortality and participation rates in patients with heart failure (HF) [4, 5]. However, this does not reflect the situation of the majority of patients, most of whom are ambulatory and cared for by general practitioners (GPs). Only a few studies have assessed the effect of HF management programmes delivered in the primary care setting [6–10]. Others recruited patients in primary care but the intervention was delivered by practice nurses or doctors’ assistants [11–13]. However, these studies do not reflect the ‘real-life’ situation of primary care in France, where practice nurses are rare at GP clinics. Therefore, more evidence is needed on the effect of patient education programmes delivered by GPs. As GPs are the doctors closest to patients, we hypothesised that a patient education delivered by them could improve the quality of life of patients with HF.

The *Education Thérapeutique des patients Insuffisants Cardiaques* (ETIC; Therapeutic Education for Patients with Cardiac Failure) trial was designed to assess whether a pragmatic educational programme for patients with CHF delivered by trained GPs could improve the quality of life of patients with CHF compared with routine care.

## Methods

### Study design and randomisation

**Fig. 1:**
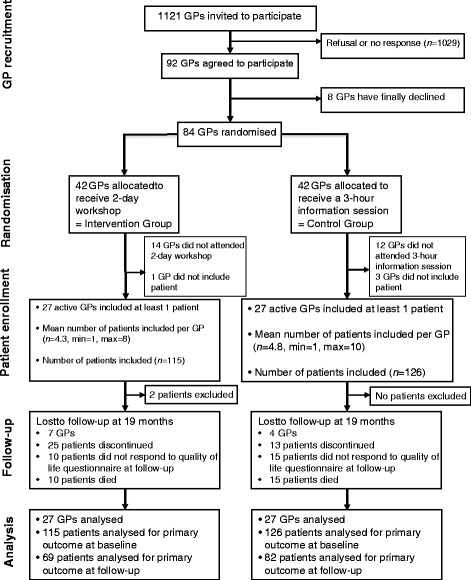
General practitioner and patient flow chart. GP, general practitioner; Min, minimum; Max, maximum

The study design has been published previously [14]. The ETIC was a cluster randomised controlled clinical trial with general practices as the unit of randomisation (Fig. 1). The trial was carried out in the four areas of the Auvergne region of France, with stratification on each. The trial is reported according to the extended CONSORT statement for cluster randomised trials. The ETIC was registered with ClinicalTrials.gov (Registration Number: NCT01065142) [15].

### Inclusion and exclusion criteria

Patients aged over 50 years, with New York Heart Association (NYHA) Stage I, II or III HF and with a reduced or preserved ejection fraction (HFrEF or HFpEF) as confirmed by the patient’s cardiologist according to the European Society of Cardiology guidelines, were eligible for inclusion [3]. Patients with NYHA Stage I HF were included because, even if asymptomatic, they had to manage the everyday manifestations of disease and might benefit from patient education; also, it was interesting to know whether the intervention had an impact on the evolution of NYHA HF stages. In contrast, the condition of patients with NYHA Stage IV HF seemed too advanced for educational sessions to have an impact on their quality of life, because patients were not included after a hospital discharge but were included in general practice with stable CHF. HFrEF was defined as an ejection fraction of ≤40 %, whereas HFpEF was defined as an ejection fraction of >40–50 % in combination with signs and/or symptoms of CHF and evidence of diastolic dysfunction (abnormal left-ventricular relaxation or diastolic stiffness) [3].

Patients with severe cognitive disorders according to the GP’s judgement, those institutionalised at the time of inclusion, those with NYHA Stage IV HF, those participating in another clinical trial and those lacking French language skills were excluded.

### Instruments and outcomes

The primary outcome was patients’ quality of life, as measured by the MOS 36-Item Short Form Health Survey (SF-36), a widely used generic instrument [16], and the Minnesota Living with Heart Failure Questionnaire (MLHFQ), an HF-specific instrument, both of which are considered good psychometric properties [17, 18]. The SF-36 questionnaire consists of eight dimensions: physical function, role physical, body pain, general health, vitality, role emotional, mental health and social function. The SF-36 physical health score incorporates physical function, role physical, body pain and general health. The SF-36 mental health score incorporates vitality, role emotional, mental health and social function. SF-36 scores range from 0 to 100: 0 indicates the worst quality of life and 100 the best. MLHFQ score ranges from 0 to 105: 0 indicates best quality of life. Quality of life was assessed at baseline and at 7, 13 and 19 months using self-administered questionnaires completed by patients or their main caregiver within 7 days of their appointment with the GP. If the patient had literacy difficulties, the main caregiver interviewed the patient and filled out the questionnaire.

The secondary outcomes were: all-cause and HF-associated mortality; all-cause and HF-associated hospitalisations and the number of days spent in hospital; cumulative number of deaths of all causes or HF-associated hospitalisations and cumulative number of days of hospitalisation; cumulative number of cases of acute HF (an acute episode reported by the GP with or without hospitalisation); cumulative number of visits to a cardiologist and cumulative number of additional GP visits (in addition to those dedicated to the trial); adherence to therapy (using a self-administrated questionnaire at baseline and at the end of follow-up) [19]; evolution of NYHA HF stage; and changes in weight and body mass index at 19 months.

### Intervention

No medication was tested in this trial and GPs were allowed to adapt patients’ treatments if necessary. The GPs in the Intervention Group received a 2-day interactive workshop that trained them to deliver a patient education programme (Table 1). The intervention consisted of patient education on standardised topics: clinical alarm signs, physical activity, diet and cardiovascular risk factors. The GPs were trained to manage their own education objectives (e.g. diet, treatment adherence) and patients’ objectives (e.g. to be able to walk their grandchildren to school). Several patient education sessions were simulated during the 2-day workshop.

**Table 1:** Training seminar for general practitioners: 2-day workshop

Module 1: Introduction	Introduction to the concepts of the *Education Thérapeutique des patients Insuffisants Cardiaques* (ETIC; Therapeutic Education for Patients with Cardiac Failure) trial and patient education
Module 2: Heart failure	Chronic heart failure: definitions; epidemiology; clinical diagnosis; treatment guidelines; echocardiographic criteria; cardiac biomarkers—B-type natriuretic peptide (BNP) and NT-proBNP (how and when to prescribe them) Clinical symptoms: how to recognise heart failure in daily practice New York Heart Association (NYHA) stages: definitions; assessment of NYHA stages from case vignettes Suspicious clinical signs Adaptation of physical activity as a function of NYHA stage
Module 3: Concepts of patient education	Assessment and building on patients’ existing knowledge Identification of lifestyle and dietary habits, physical activity, hobbies, leisure activities, projects and resources available to the patient Assessment of patients’ stage of change, motivation and attitude Collaboration with the patient to define achievable and measurable objectives
Module 4: Communication	Communication skills Communication tools Motivational interviewing Lifestyle counselling based on the Five As model (ask, assess, advise, assist, and arrange)
Module 5: Role play to simulate a patient consultation with the general practitioner	Identification and use of patients’ knowledge (clinical alarm signs, physical activity, diet and cardiovascular risk factors), values, motivation, projects and resources to involve the patient in their personal objectives Classification of these personal objectives by therapeutic priority and patient preference Use of effective communication strategies
Module 6: Case report forms	Inclusion and exclusion criteria How to promote and present the ETIC trial to patients How to fill in the case report forms How to organise the follow-up and topics: educational booklet and educational tools (i.e. dietary leaflets, clinical alarm signs)

General practitioners were trained to deliver a patient education programme during a 2-day interactive workshop (six modules)

The patients’ education sessions were performed by their GPs and are detailed in the trial protocol [14]. The education sessions were standardised in their timing (every 3 months) and the topics covered. The GPs received an education booklet with the topics covered and education tools (i.e.; Table 2 and Additional file 1: Dietary leaflets and information on clinical alarm signs). The first educational session (educational diagnosis) for patients occurred during Month 1 and covered several topics: lifestyle and dietary habits, physical activity, hobbies, leisure activities, projects and details of resources available to patients (Additional file 2: The educational diagnosis summary). This first step was necessary to establish patients’ knowledge, attitude and motivation. Patients had a further four education sessions, at 4, 7, 10 and 13 months, followed by an overview session at 19 months. The patient education sessions were adapted to each patient, on the basis of the first education session at Month 1 and at each of the following visits, to match the needs and motivation of each patient. At the end of each visit, the patients fixed and agreed their own personal objectives with the GP (Additional file 3: The education sessions summary). The healthcare providers had the flexibility to adapt the programme according to the patients’ experience, knowledge, needs and desires [20, 21]. Consequently, education sessions were simultaneously standardised in their topics and their timing (every 3 months) and personalised to individual patients. To assess the quality of the intervention, at the end of each education session the GPs reported what they did and the topics discussed (e.g. clinical alarm signs, physical activity, diet, cardiovascular risk factors and adherence; Additional file 3: The education sessions summary).

**Table 2:** Education intervention topics

Knowledge	Do you suffer from heart failure?
Attitudes	What is ‘heart failure’ for you?
Motivation	What do you know about heart failure?
	How do you live with this disease?
	What impact has heart failure had on your life (personal, professional, social)?
	What are your fears?
	What are your expectations?
Clinical alarm signs	For you, what could be a clinical alarm sign of your heart failure?
	What should you do to detect clinical alarm signs?
	Do you know what to do if you detect clinical alarm signs?
Physical activity	What does physical activity mean for you?
	What physical activities do you undertake? Housework? Leisure (e.g. gardening)? Transportation (e.g. walking, car)?
	When are you breathless? (New York Heart Association assessment)
	Regarding your habits, what would you be ready to change?
Diet	Where do you eat your meals?
	Who does the cooking?
	High-salt food: what do you know about it? How much do you consume?
	What is your point of view and what changes are you ready to make?
	For those with a body mass index ≥30: what are your diet mistakes (snack food, overeating) or diet troubles?
	For those with a body mass index ≤18 (adult patients) or 21 (elderly patients): what are your diet mistakes or diet troubles?

The general practitioners received an education booklet covering the following topics: knowledge/attitudes/motivation; clinical alarm signs; physical activity; and diet. There was no predetermined order – each theme was evoked depending on patients’ needs and based on the first education session

### Control

GPs in the Control Group attended a 3-hour information session to learn about the case report forms and the inclusion and exclusion criteria. Their patients had the same schedule for visits as those in the Intervention Group but without a specific education intervention (i.e., at 1, 4, 7, 10, 13 and 19 months).

### Statistical considerations

The sample size estimation and statistical analyses were presented in Vaillant-Roussel et al. [14]. Sample size estimation was performed to detect a difference of 12 points for quality of life outcomes (SF-36 and MLHFQ), which corresponds to an effect size of 0.6, with a statistical power of 90 % and a two-sided Type 1 error of 5 %, taking into account clustering by practice (intra-cluster correlation was considered to be between 0.1 and 0.2) [13, 16, 17, 22, 23] A 20 % dropout rate was assumed. On the basis of several simulations, it was estimated that 40 GPs in general practices recruiting five patients each were required per group, resulting in the recruitment of 200 patients in each group. The statistician was blinded with regard to treatment allocation.

Statistical analyses were realised in intention to treat using Stata (version 13; StataCorp LP, College Station, TX, USA). The main analysis was performed with hierarchical linear regression models to estimate the effects of the intervention on SF-36 and MLHFQ scores for the post-baseline time points adjusted for the baseline score, as proposed previously [24]. Random effects were used for practice, individuals within practices and repeated measurements per individual (slope and intercept). The results were expressed as the regression coefficient (b) and 95 % confidence interval (CI). Intra-class correlation coefficients (ICCs) were presented by group. Finally, a sensitivity analysis was used to investigate the nature of the missing data and a per-protocol analysis was also performed.

## Results

### Recruitment of general practitioners and patients

An overview of the recruitment of GPs and patients is presented in Fig. 1. Overall, 54 (64 %) of the randomised GPs were active and enrolled at least one patient into the trial. The inclusion period lasted 1 year. The GPs recruited 243 patients. Two patients with NYHA Stage IV HF were excluded from the analysis.

### Baseline characteristics of general practitioners and patients

The characteristics of the active GPs were comparable between the Intervention and Control Groups (Table 3). The characteristics of the 241 patients remaining in the trial are shown in Table 4; 115 patients were included in the Intervention Group and 126 in the Control Group. Their mean age was 74 years (± 10.5) and 62 % were men. HF had been diagnosed between 0 and 35 years previously and at a median of 5 years earlier (inter-quartile range = 2–10). There were 101 patients in the Intervention Group (87.8 %) and 101 patients in the Control Group (80.2 %) with HF of NYHA Stages II or III (*P* = 0.11). The mean left-ventricular ejection fraction was 50.9 % (± 13.2 %) in the Intervention Group and 47.7 % (± 15.2 %) in the Control Group (*P* = 0.16). Patients with HFpEF in the Intervention and Control Group numbered 93 (80.9 %) and 94 (74.6 %), respectively (*P* = 0.24).

**Table 3:** Baseline characteristics of 54 general practitioners

	Intervention Group (*n* = 27)	Control Group (*n* = 27)
Gender male, *n* (%)	17 (63)	20 (74.1)
Age (years), mean (SD)	50.2 (7.9)	51.6 (7.3)
Length of time in practice (years), mean (SD)	21.9 (7.9)	23.5 (8)
Type of practice, *n* (%)		
Rural	4 (14.8)	3 (11.2)
Suburban	16 (59.3)	12 (44.4)
Urban	7 (25.9)	12 (44.4)
Group practices, *n* (%)	16 (59.3)	19 (70.4)
Trainee supervisors^a^ *n* (%)	19 (70.4)	13 (48.2)
Number of patients included, mean (SD)	4.3 (2)	4.8 (1.8)

^a^Trainee supervisors were general practitioners (GPs) involved in teaching, GPs who were university lecturers or those who received students for internship; *SD* standard deviation

**Table 4:** Baseline patient characteristics

	Intervention (*n* = 115)	Control (*n* = 126)	*P*-value
Gender male, *n* (%)	69 (60)	80 (63.5)	0.58
Age (years), mean (SD)	74.7 (10.3)	73.5 (10.8)	0.42
Chronic heart failure duration, median (IQR)	5 (1–11)	5 (2–10)	0.66
EF mean (SD)	50.9 (13.2)	47.7 (15.2)	0.16
HFpEF *n* (%)	93 (80.9)	94 (74.6)	0.24
NYHA stage, *n* (%)			
I	14 (12.2)	25 (19.8)	
II	69 (60)	67 (53.2)	0.26
III	32 (27.8)	34 (27)	
Current smoker, *n* (%)	14 (12.2)	25 (19.8)	0.11
BMI kg/m^2^, *n* (%)^a^			
< 25	25 (22.2)	44 (35.8)	
25–30	44 (38.9)	52 (42.3)	**0.008**
≥ 30	44 (38.9)	27 (21.9)	
Hypertension, *n* (%)	72 (62.6)	65 (51.6)	0.08
Type 2 diabetes, *n* (%)	30 (26.1)	22 (17.5)	0.10
Hypercholesterolaemia, *n* (%)	50 (43.5)	54 (42.9)	0.92
COPD, *n* (%)	9 (7.8)	19 (15.1)	0.08
SF-36 mental health score, mean (SD)	60.3 (21.2)	60.1 (21.3)	0.89
SF-36 physical health score, mean (SD)	52.1 (22.5)	50.9 (22.1)	0.66
MLHFQ score, mean (SD)^b^	29.1 (22.1)	24.4 (21.7)	0.07
<24	50 (52.6)	64 (57.7)	
24–45	22 (23.2)	26 (23.4)	0.64
>45	23 (24.2)	21 (18.9)	
Patient adherence^c^, *n* (%)			
Good adherence	46 (44.2)	49 (43)	
Minor nonadherence	56 (53.9)	60 (52.6)	0.67
Nonadherence	2 (1.9)	5 (4.4)	
	Intervention (*n* = 102)	Control (*n* = 121)	*P*-value
Treatment, *n* (%)^e^	98 (96.1)	117 (96.7)	1.00
β-blocker	63 (61.8)	73 (60.3)	0.83
ACE inhibitor	51 (50)	65 (53.7)	0.58
ARB	34 (33.3)	29 (24)	0.12
ACE inhibitor or ARB^d^	85 (83.3)	93 (76.9)	0.23
β-blocker and (ACE inhibitor or ARB)	56 (54.9)	60 (49.6)	0.43
Thiazide diuretics	12 (12.6)	16 (13.2)	0.74
Loop diuretics	71 (69.6)	78 (64.5)	0.42
Thiazide diuretics or loop diuretics	76 (74.5)	90 (74.3)	0.98
Mineralocorticoid receptor antagonists	15 (14.7)	21 (17.4)	0.59
Digoxin	11 (10.8)	10 (8.3)	0.52

*ACE inhibitor* angiotensin-converting enzyme inhibitor, *ARB* angiotensin receptor blocker, *COPD* chronic obstructive pulmonary disease, *HFpEF* heart failure with preserved ejection fraction, *EF* ejection fraction, *SD* standard deviation

^a^ *n* = 5 missing data for body mass index. ^b^Questionnaires with more than three missing responses were excluded (*n* = 35: 15 in the Control Group and 20 in the Intervention Group). ^c^ *n* = 23 missing data for adherence. ^d^One patient had angiotensin-converting enzyme inhibitor and angiotensin receptor blocker

^e^18 patients had missing data concerning treatments at baseline

Significant *P*-value are in bold

There was no difference between the Intervention and Control Groups with regard to treatments and patient adherence (Table 4); 4 % of the patients received no treatment at baseline. There was no difference in quality of life scores between the Intervention and Control Groups when treatment was stratified according to HFrEF and HFpEF (data not shown). There was no difference between the two groups with regard to quality of life (detailed in Table 4). The correlation coefficients of the MLHFQ and SF-36 physical health scores and the MLHFQ and SF-36 mental health scores were −0.63 and −0.64, respectively.

### Primary outcomes

Quality of life scores are presented in Table 5. The ICC associated with the MLHFQ primary outcome at 19 months was 0.11. Changes from the baseline were analysed in the Intervention and Control Groups only for patients whose data were available at the end of the trial. The regression coefficient, adjusted for the baseline results, of the MLHFQ score was b = 1.19 (95 % CI: −5.94–8.32, *P* = 0.74). The differences in MLHFQ score in the Intervention and Control Groups at the 19-month follow-up were 3 (−4–13) and 1 (−5–13), respectively. There was no difference in MLHFQ score during follow-up at 7 and 13 months, respectively: 33.9 (± 29.2) in the Intervention Group versus 28.3 (± 22.5) in the Control Group at 7 months, *P* = 0.14; 33.4 (± 22.1) in the Intervention Group versus 29.5 (± 24.3) in the Control Group at 13 months, *P* = 0.22.

**Table 5:** End points at Month 19

	Intervention (*n* = 69)	Control (*n* = 82)	*P*-value
Primary outcomes			
SF-36 mental health score, mean (SD)	58 (22.1)	58.7 (23.9)	0.57
SF-36 physical health score, mean (SD)	52.8 (23.8)	51.6 (25.5)	0.58
MLHFQ score, mean (SD)	33.4 (22.1)	27.2 (23.3)	0.74
Secondary outcomes			
NYHA stage, *n* (%)^a^			
I	14 (22.6)	21 (29.2)	
II	35 (56.5)	34 (47.2)	0.73
III	12 (19.3)	15 (20.8)	
IV	1 (1.6)	2 (2.8)	
BMI kg/m^2^, *n* (%)^b^			
< 25	13 (22)	29 (40.3)	
25–30	25 (42.4)	33 (45.8)	**0.007**
≥ 30	21 (35.6)	10 (13.9)	
Patient adherence^c^, *n* (%)			
Good adherence	23 (37.1)	32 (42.1)	
Minor nonadherence	35 (56.5)	42 (55.3)	0.55
Nonadherence	4 (6.4)	2 (2.6)	
Mortality, *n* (%)	10/115 (8.7)	15/126 (11.9)	0.41
Total CHF decompensation/visits (%)	65/470 (13.8)	93/545 (17.1)	0.16
Hospitalisation for CHF decompensation/visits (%)	18/65 (27.7)	22/93 (23.7)	0.57
Hospitalisation not for CHF decompensation/visits (%)	50/470 (10.6)	59/545 (10.8)	0.92
Hospitalisation/visits (%)	62/470 (13.2)	74/545 (13.6)	0.86
Hospitalisation/patients (%)	41/115 (35.7)	54/126 (42.9)	0.26
Total number of days of hospitalisation	1037	867	
HF hospitalisation/patients (%)	13/115 (11.3)	17/126 (13.5)	0.61
Death or hospitalisation/patients (%)	45/115 (39.1)	60/126 (47.6)	0.18
Death or HF hospitalisation/patients (%)	20/115 (17.3)	28/126 (22.2)	0.35
^d^Total visits related to GP/patients (%)	90/115 (78 %)	106/126(84 %)	0.24
^d^Number of GP visits/patient, mean (SD)	8.1 (5.3)	6.4 (4.5)	**0.02**
Total visits related to cardiologist/patients (%)	85/115 (74 %)	84/126 (67 %)	0.22
Number of cardiologist visits/patient, mean (SD)	3.1 (2.2)	3.1 (2)	0.92

^a^ *n* = 17 missing data for New York Heart Association stage

^b^ *n* = 20 missing data for body mass index

^c^ *n* = 13 missing data for adherence

^d^Additional general practitioner visits (in addition to those dedicated to the study)

Significant *P*-value are in bold

The ICC associated with the SF-36 mental and physical health primary outcome at 19 months was similar (ICC = 0.01). The regression coefficients, adjusted for the baseline results, of the SF-36 mental and physical health scores were b = −1.7 (−7.6–4.15; *P* = 0.58) and b = 1.6 (−4.03–7.21; *P* = 0.57), respectively. Differences from the baseline in SF-36 mental and physical scores between the Intervention and Control Groups at the 19-month follow-up were: −3.2 (−14.5–4.7) and −0.08 (−13.6–7.5); and −1 (−8–8) and 0 (−12–10), respectively.

**Fig. 2:**
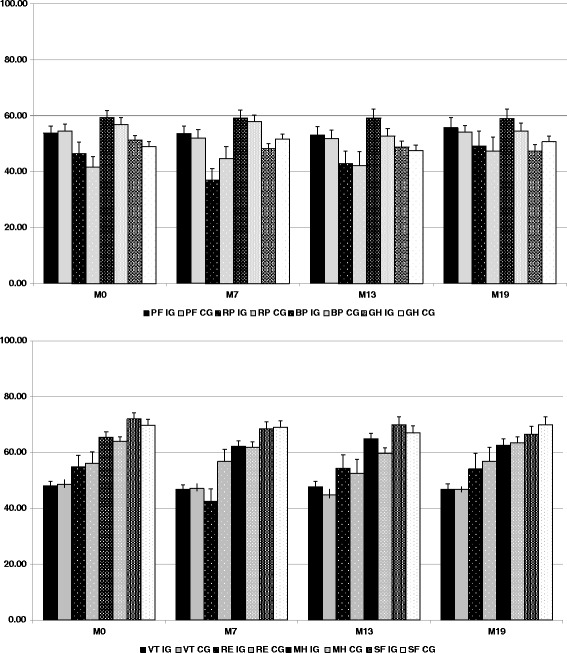
Changes in quality of life from baseline to the follow-up period for each Short Form 36 variable. IG, Intervention Group; CG, Control Group; Short form 36 physical health variables: PF, physical function; RP, role physical; BP, body pain; GH, general health. Short form 36 mental health variables: VT, vitality; RE, role emotional; MH, mental health; SF, social function. Short Form 36 variables are described at baseline (M0) and at 7, 13 and 19 months (M7, M13 and M19)

Figure 2 shows the change in quality of life from baseline to the follow-up period for each SF-36 variable (*P* = not significant between the Intervention and Control Groups). A subgroup analysis, based on age classes, asymptomatic (I) and symptomatic (II and III) NYHA stages, HFrEF and HFpEF, demonstrated no significant difference in quality of life scores.

### Secondary outcomes

**Fig. 3:**
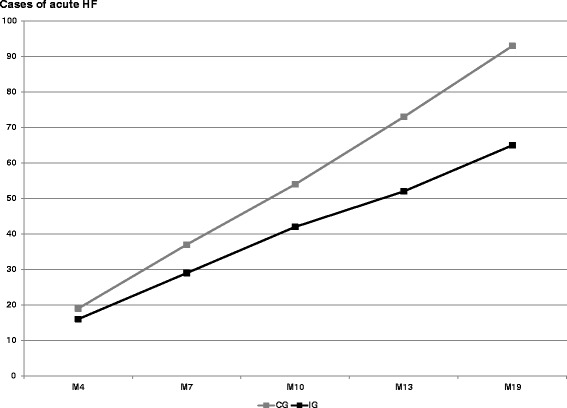
Cumulative number of patients with acute heart failure. IG, Intervention Group; CG, Control Group; HF, Heart Failure. Assessment at 4, 7, 10, 13 and 19 months (M4, M7, M10, M13 and M19). A case of acute heart failure was defined as an acute episode reported by the general practitioner with or without hospitalisation

Mortality and healthcare outcomes are detailed in Table 5. Fifteen deaths occurred in the Control Group and 10 in the Intervention Group, although this difference was not statistically significant (*P* = 0.41). The cumulative number of cases of acute HF was 158/1015 visits (15.6 %; 65/470 for the Intervention Group versus 93/545 for the Control Group, *P* = 0.16; Table 5 and Fig. 3). Of all patients with acute HF, 40/158 (25.3 %) were hospitalised. When patients were hospitalised for any cause, the cumulative number of days in hospital was 1037 days in the Intervention Group (median: 8 days [range: 4–23]) and 867 days in the Control Group (median: 8 days [range: 3–25]; *P* = 0.58).

**Fig. 4:**
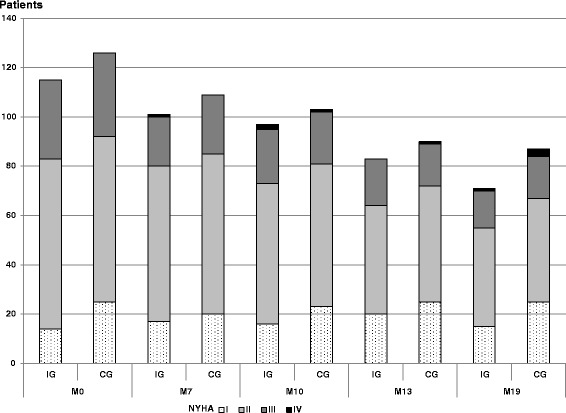
Evolution of New York Heart Association stages during follow-up. IG, Intervention Group; CG, Control Group. New York Heart Associations stage assessment at baseline (M0) and at 7, 13 and 19 months (M7, M13 and M19)

During the 19-month follow-up period, 25/115 (22 %) patients in the Intervention Group and 20/126 (16 %) patients in the Control Group had no additional GP visits (in addition to those dedicated to the trial; *P* = 0.24). Among patients who consulted their GP, the mean number of GP visits was 8.1 (± 5.3) in the Intervention Group and 6.4 (± 4.5) in the Control Group (*P* = 0.02). There was no difference in the number of visits to a cardiologist (Table 5). Figure 4 describes the evolution of NYHA stages during the follow-up period.

## Discussion

### Summary of the main results

A pragmatic patient education intervention for HF delivered by trained GPs did not improve patients’ quality of life compared with routine care. There was no difference between the groups in MLHFQ (*P* = 0.74), SF-36 mental health (*P* = 0.57) or SF-36 physical health (*P* = 0.58) questionnaire scores at the 19-month follow-up examination. The ICC associated with MLHFQ score was 0.11 and those associated with the SF-36 mental and physical health scores were similar (ICC = 0.01).

### Meaning of the findings

Although this trial did not detect any impact on the primary outcome, it is the first to examine data derived from patients enrolled, treated and followed-up in primary care [7–10]. In Europe, most published studies on patient education programmes involve hospitalised patients or patients discharged from hospital. The profiles of these patients differ from those treated in primary care, which comprise a population with stable HF, as in the ETIC, composed of elderly patients with a relatively good quality of life [12, 25]. The characteristics of patients with HF enrolled in the ETIC more closely resemble those of patients enrolled in the French IMPROVEMENT study on primary care, where the mean patient age was 73 years and 40 % of patients were female [26]. Many published studies include only patients with HFrEF [12], younger patients [7, 9], or predominantly male patients [8, 11]. Consequently, most data published to date relate to patients with HFrEF [27]. The ETIC chose a pragmatic design and included a broad range of patients with CHF, most of whom had HFpEF, because we deliberately chose not focus on just one segment of the CHF population [27]. In the IMPROVEMENT study, only 51 % of patients with an echocardiogram exhibited left-ventricular systolic dysfunction (poorly contracting left ventricle, enlarged left ventricle or ejection fraction under 40 %) [26].

The quality of life of the patients was measured using two questionnaires, the MLHFQ and the SF-36, because the first is specific and the second is generic. The sample size was estimated by taking into account an anticipated ICC of between 0.1 and 0.2 [22]. According to the ICC results, the data were more dispersed for MLHFQ score (ICC = 0.11) than SF-36 scores (mental SF-36 and physical SF-36 ICC = 0.01), which indicates that, in this context, the MLHFQ is probably more discriminative. These results could be useful for future studies in similar settings.

The quality of life scores at the end of follow-up at 19 months appeared surprisingly stable in the elderly patients enrolled in the ETIC study, especially the SF-36 physical health score, reflecting the natural progression of health-related quality of life in general population [28]. We cannot attribute this to an effect of our education sessions, because this stability in quality of life was found in both groups. We propose that the act of participating in a study stabilised patients’ quality of life (an example of the Hawthorne effect). The same stability was found in another study involving patients with stable CHF conducted in primary care in Germany [12].

We compared the number of additional GP consultations for all patients and observed no difference between the Intervention and Control Groups. However, among patients who consulted their GP, the mean number of consultations was significantly higher in the Intervention Group. This was not the case for visits to the cardiologist. These results are consistent with another study in the primary care setting [12].

### Strengths and limitations of the trial

The ETIC was one of the largest trials in the primary care setting to study the effects of an educational intervention on patients with CHF. A cluster design was chosen for pragmatic reasons and to avoid contamination bias.

Reviews of studies on management programmes for patients with HF have shown mixed effects on hospital admissions, mortality and quality of life [29, 30]. There was large variability in the complexity of case management, patient education, training of care managers and care settings. Overall positive effects on predominantly disease-specific quality of life were found in a short-term follow-up but the results observed during longer follow-ups were largely non significant. Short-term positive effects on quality of life were observed in hospitalised patients and those with acute HF, who exhibited low baseline scores, enabling short-term effects to be detected in comparison with controls [7, 10]. The potential for improving the quality of life of patients recovering from hospitalisation may be higher than that of patients with stable disease treated in primary care [13]. The ETIC trial included patients with stable HF with relatively high baseline quality of life scores; perhaps it was unrealistic to attempt to improve the quality of life of this population, even if the follow-up period (19 months) was longer than in other studies.

The aim of this trial was a change of 12 points in the quality of life scores, to show not only a statistically significant but also a clinically relevant difference [31]. Although some studies tried to find a difference of five points in quality of life scores, others chose a difference of 12 points for the same reasons [12, 13, 32]. However, this difference cannot explain the absence of an effect. It is also the case for the power, because even if a difference of five points had been chosen, this study would not show a significant difference (Table 5).

Finally, the intensity of the intervention delivered by the GPs may have been too low and other factors outside the disease-related intervention may have had a greater impact on quality of life. Quality of life is a multifactorial measure that may be too complex to be changed solely by GPs trained in patient education. Consequently, even if quality of life is a good clinical indicator of health status, it is probably difficult to show a significant improvement as the result of an intervention in an elderly population with stable HF. Rather, utilisation of healthcare and treatment optimisation or self-care behaviour may be more effective, measurable outcomes [12, 32–34].

The number of participants per site may seem inadequate but we sought the best balance between the number of GPs and the number of patients enrolled by each GP based on the capacity for inclusion and feasibility in terms of the workload (including the follow-up). Another team in Germany estimated the same capacity for inclusion per GP [12]. Finally, it is important to note that each active GP contributed 4.3 (± 2) patients to the Control Group and 4.8 (± 1.8) patients to the Intervention Group whereas, according to the study design, the number of patients to be included in each group should have been five.

The limitations of this trial include a dropout rate of 36 % after randomisation among GPs, either because they withdrew consent to participate (31 %) or failed to recruit patients (5 %). Although the recruitment goal was not reached, the lack of significant difference between the randomised groups cannot be attributed to a lack of power: the effect size for the primary outcome was minimal (less than 0.27 [−0.07–0.61]) and the ICC was lower than expected, meaning that the sample size could have been smaller. In France, clinical research by GPs in primary care is still relatively new and this is one possible explanation for the high dropout rate [35]. Another explanation could be that GPs who agreed to participate but ultimately did not found the trial workload to be too heavy. The generalisability of the data from the remaining GPs who participated in the ETIC trial can be considered good, because they are very similar to the national characteristics of GPs as assessed in 2009 [36]. Furthermore, the characteristics of the patients included were similar to those of patients with CHF in France in primary care [26].

At baseline, the patients’ characteristics were similar, except that those in the Intervention Group were more likely to be overweight or obese compared with those in the Control Group. The same difference was found at the end of follow-up. The patients had a similar quality of life according to the SF-36 but, although without statistical significance, MLHFQ scores were worse in the Intervention Group (*P* = 0.07). One possible explanation is that selection bias was present: the training received by the GPs in the Intervention Group may have made them feel more competent and, therefore, they may have included more severely ill patients in the trial. To avoid this bias, we could have randomised the GPs after they had recruited their patients, using Zelen’s method [37]. However, this option was not feasible because of the short life expectancy of the patients: we considered the mean age of patients at the time of inclusion to be high, at 74 years. It was inadvisable to recruit patients over a period longer than 1 year and then randomise the GPs to receive training.

As the ETIC population was a population with a good quality of life and with a significant proportion of patients with HFpEF, our results could not be extrapolated to patients with HFrEF and a poor quality of life. Most of the patients in our study had HFpEF and, as treatment is not conclusively known to be of benefit in such patients, we hypothesise that it is the same with patient education. However, when outcomes were stratified according to HFrEF and HFpEF, the type of HF had no influence. We failed to demonstrate an impact of our intervention regardless of the type of HF.

To assess the quality of the intervention, at the end of each education session the GPs reported what they did and the topics discussed [14]. However, we cannot make any inferences on the intensity of the intervention delivered by the GPs in the Intervention Group. It is possible that a 2-day workshop is insufficient to teach GPs how to conduct successful counselling of HF patients regarding lifestyle. However, a longer workshop may be unrealistic for GPs and would not equate to a pragmatic design suitable for everyday practice. Finally, we cannot exclude the possibility that we were unable to observe an effect of the intervention because of the inclusion of motivated GPs with a special interest in the topic in both trial arms.

## Conclusion

Patient education delivered by GPs to elderly patients with stable CHF in the ETIC programme did not achieve an improvement in their quality of life compared with routine care. Further research on improving the quality of life and clinical outcomes of elderly patients with HF in primary care is necessary.

## Abbreviations

BMI, body mass index; CHF, chronic heart failure; CI, confidence interval; GP, general practitioner; HF, heart failure; HFpEF, Heart failure with preserved ejection fraction; HFrEF, Heart failure with reduced ejection fraction; ICC, intra-cluster coefficient; MLHFQ, Minnesota Living with Heart Failure Questionnaire; NYHA, New York Heart Association; SF-36, MOS 36-Item Short Form Health Survey.

## Additional files

Additional file 1: Dietary leaflets and information on clinical alarm signs. (PPT 11110 kb) Additional file 2: The educational diagnosis summary. The educational diagnosis was the first educational session and explored lifestyle and dietary habits, physical activity, hobbies, leisure activities, projects and resources available for patients. (DOC 35 kb) Additional file 3: The education sessions summary. Patients had a further four education sessions, at 4, 7, 10 and 13 months, followed by an overview session at 19 months. At the end of each visit, the patients fixed and agreed their own personal objectives with the GP. (DOC 32 kb)
