# Supplementary material for: Impact of patient education on chronic heart failure in primary care (ETIC): a cluster randomised trial
Source: BMC Fam Pract. 2016 Jul 19;17:80. doi: 10.1186/s12875-016-0473-4 (PMC4949928; doi:10.1186/s12875-016-0473-4)
Supplement: Additional file 1: — Dietary leaflets and information on clinical alarm signs. (PPT 11110 kb) [file 12875_2016_473_MOESM1_ESM.ppt]

## Slide 1
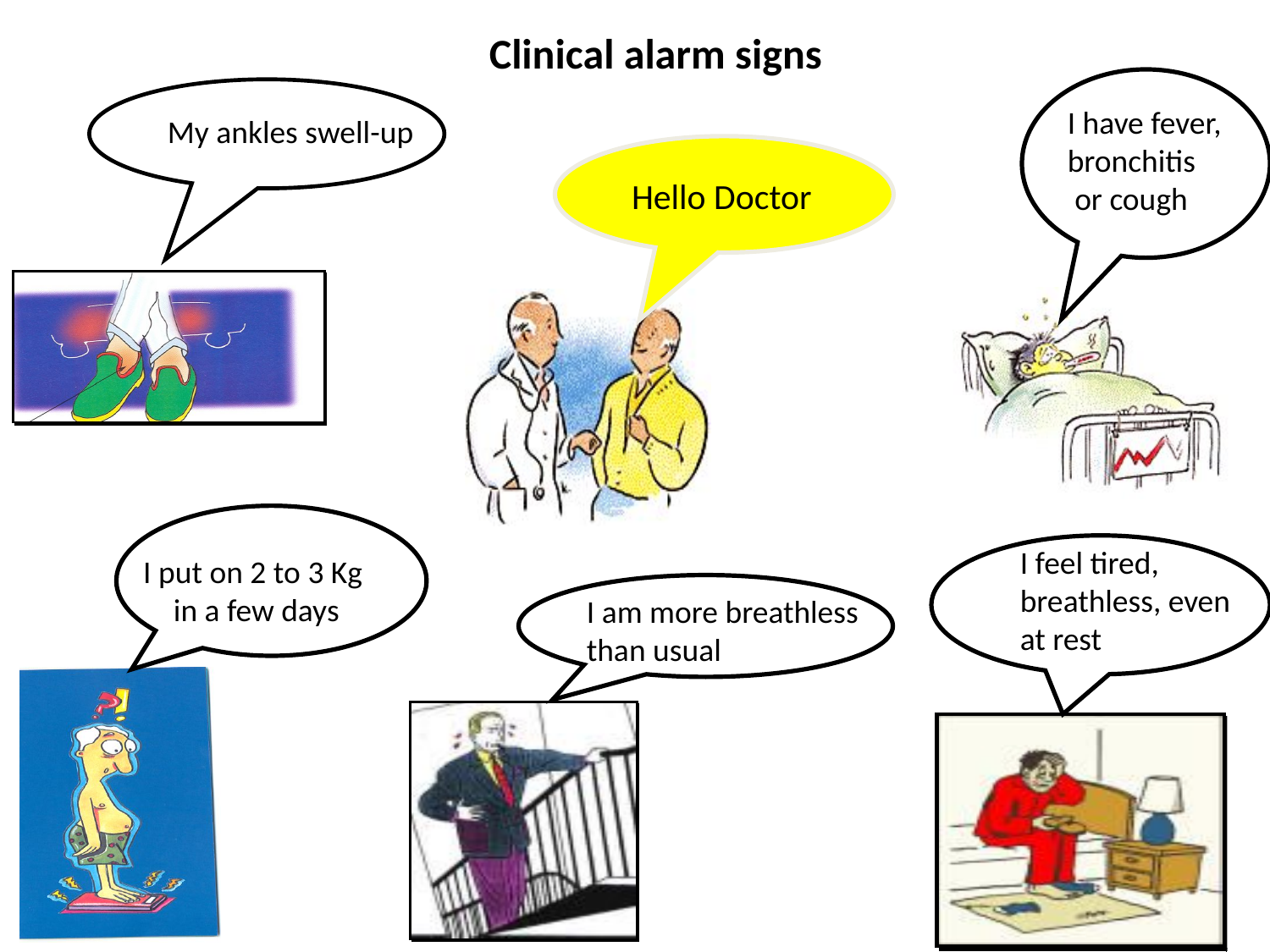

Clinical alarm signs
I have fever,
bronchitis
 or cough
My ankles swell-up
 Hello Doctor
I feel tired, breathless, even at rest
I put on 2 to 3 Kg
in a few days
I am more breathless than usual

## Slide 2
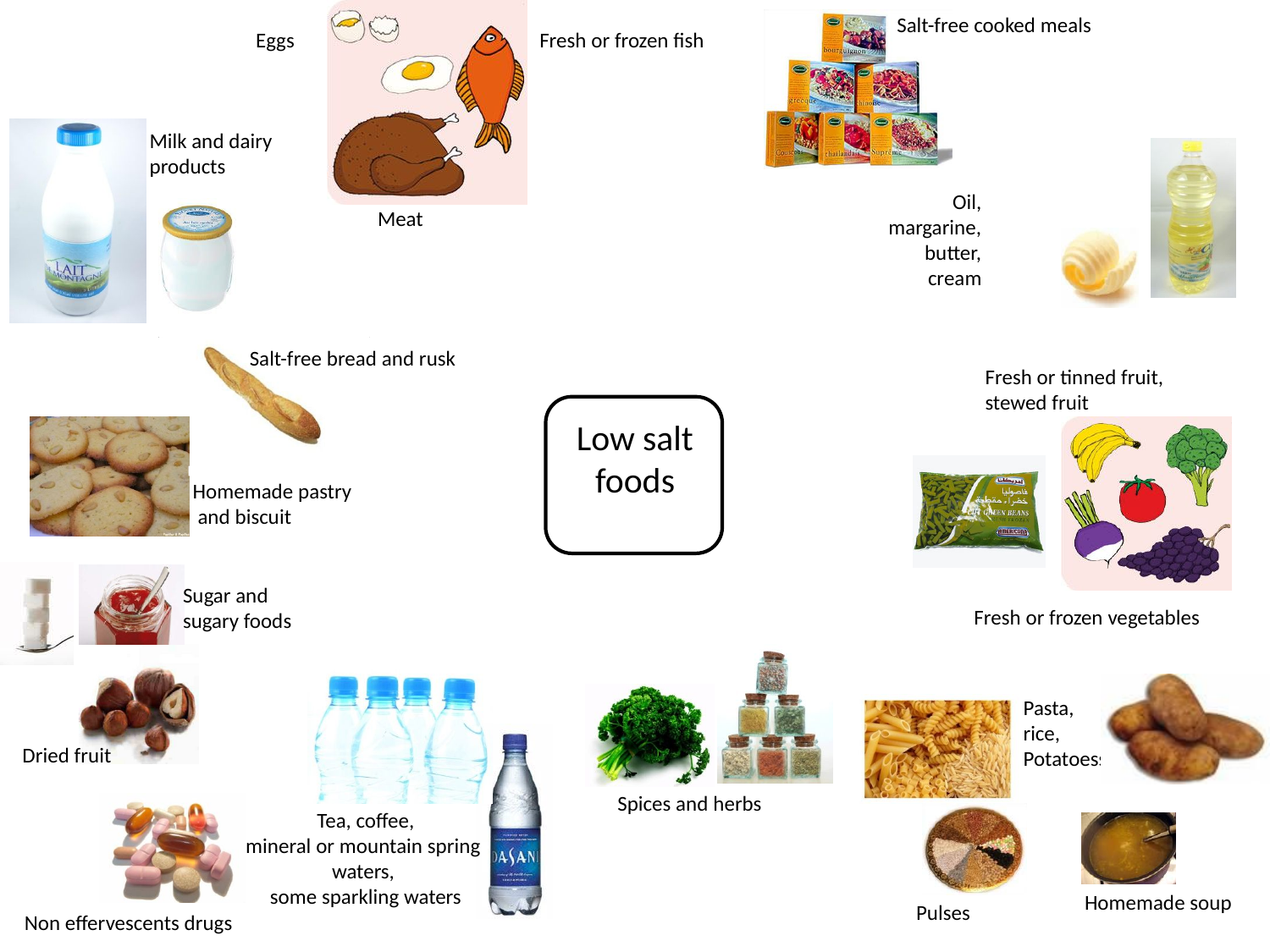

Salt-free cooked meals
Eggs
Fresh or frozen fish
Milk and dairy
products
Oil,
margarine,
butter,
cream
Meat
Salt-free bread and rusk
Fresh or tinned fruit,
stewed fruit
Low salt foods
Homemade pastry
 and biscuit
Sugar and
sugary foods
Fresh or frozen vegetables
Pasta,
rice,
Potatoess
Dried fruit
Spices and herbs
Tea, coffee,
mineral or mountain spring waters,
some sparkling waters
Homemade soup
Pulses
Non effervescents drugs

## Slide 3
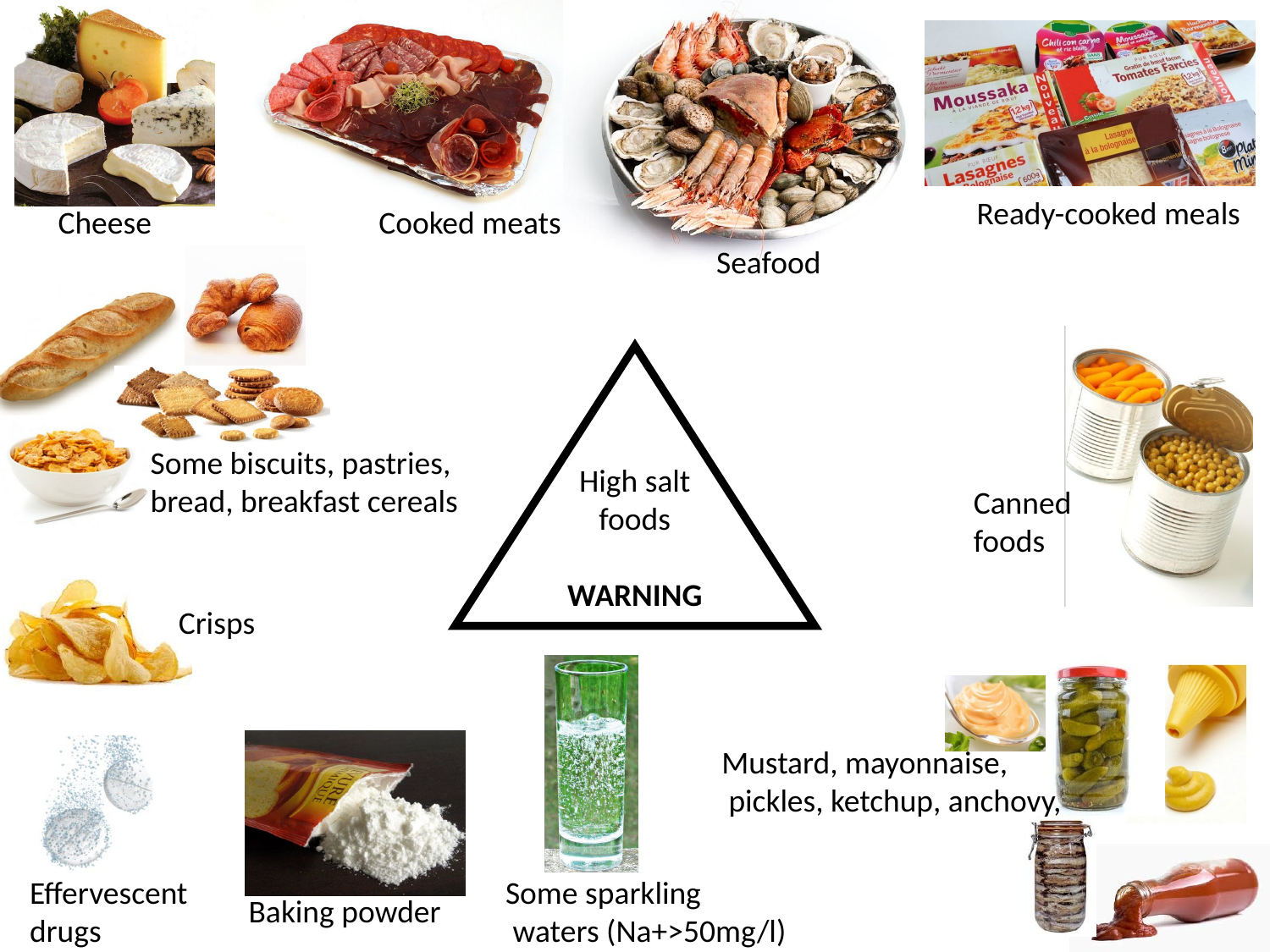

Ready-cooked meals
Cheese
Cooked meats
Seafood
High salt
foods
WARNING
Some biscuits, pastries,
bread, breakfast cereals
Canned
foods
Crisps
Mustard, mayonnaise,
 pickles, ketchup, anchovy,
Some sparkling
 waters (Na+>50mg/l)
Effervescent
drugs
Baking powder
