# Supplementary material for: Impact of patient education on chronic heart failure in primary care (ETIC): a cluster randomised trial
Source: BMC Fam Pract. 2016 Jul 19;17:80. doi: 10.1186/s12875-016-0473-4 (PMC4949928; doi:10.1186/s12875-016-0473-4)
Supplement: Additional file 2: — The educational diagnosis summary. The educational diagnosis was the first educational session and explored lifestyle and dietary habits, physical activity, hobbies, leisure activities, projects and resources available for patients. (DOC 35 kb) [file 12875_2016_473_MOESM2_ESM.doc]

| **Main care-giver** | **General practitioner** | **Cardiologist** | **Nurse** |
| --- | --- | --- | --- |
| Last name: | Last name: | Last name: | Last name: |

**First education session at one month**

Year of heart failure diagnosis: …………….

Current health problems:.....................................................................................................................

- Diabetes ❑ Dyslipidaemia ❑ Hypertension
- Tobacco: ❑ Alcohol

| **Life-style:**  Place of living:  Lives with:  Carers:  Profession (current or retired): | **Physical activity:**  Household:  Leisure (e.g. gardening):  Transportation (e.g. walking, car): |
| --- | --- |
| **Eating habits:**  Where meals are eaten:  Who cooks? :  Eats with:  Consumption of high salt foods: | **Hobbies, leisure activities, projects:**  -  -  -  -  - |
| **Knowledge, attitudes and motivation:** | |
