# Supplementary material for: Impact of patient education on chronic heart failure in primary care (ETIC): a cluster randomised trial
Source: BMC Fam Pract. 2016 Jul 19;17:80. doi: 10.1186/s12875-016-0473-4 (PMC4949928; doi:10.1186/s12875-016-0473-4)
Supplement: Additional file 3: — The education sessions summary. Patients had a further four education sessions, at 4, 7, 10 and 13 months, followed by an overview session at 19 months. At the end of each visit, the patients fixed and agreed their own personal objectives with the GP. (DOC 32 kb) [file 12875_2016_473_MOESM3_ESM.doc]

| Date :  ……………….. | Resources and levers | Difficulties |
| --- | --- | --- |
| NYHA  stage: |  |  |
| Current treatment  -  -  -  -  -  -  -  -  -  -  -  weight (Kg) :  Height (m) :  BMI (Kg/m2): |

**SYNTHESIS**

**Personal objectives**

-………………………………………

-………………………………………

-………………………………………

❑ Synthesis validated by the patient

**During the consultation, did you discuss about :**

Yes No

Adherence to treatment 1 ❑ 2 ❑

Dietary ….. 1 ❑ 2 ❑

Physical activity 1 ❑ 2 ❑

Tobacco... 1 ❑ 2 ❑

Clinical alarm signs…………………………………………..……………………………….….. 1 ❑ 2 ❑

Others : ………………………………………………………………………………………………………………………………………………………………………………………………………………………………………………………………………………………………………………………………………………………………………………………………………………………………………………………………………………………………………………………………………………………………………………
